# Analysis of sero-epidemiological characteristics of varicella in healthy children in Jiangsu Province, China

**DOI:** 10.1186/s12879-018-3496-8

**Published:** 2018-11-14

**Authors:** Lei Zhang, Wang Ma, Yuanbao Liu, Yong Wang, Xiang Sun, Ying Hu, Xiuying Deng, Peishan Lu, Fenyang Tang, Zhiguo Wang, Minghao Zhou

**Affiliations:** 1 Department of Epidemiology, School of Public Health, Nanjing Medical University, Nanjing, 211166 Jiangsu Province China; 2 Department of Expanded Programme on Immunization, Jiangsu Provincial Center for Disease Control and Prevention, Nanjing, 210009 Jiangsu Province China; 3 First Affiliated Hospital of Nanjing Medical University, Nanjing, 211166 Jiangsu Province China

**Keywords:** Varicella, Geometric antibody concentrations, Seroprevalence

## Abstract

**Background:**

In recent years, outbreaks of varicella have continued to occur, and the coverage rate of varicella vaccine in Jiangsu Province, China, remains unclear. This study aims to analyse the levels of immune antibody against varicella and obtain a comprehensive understanding of the varicella attenuated live vaccine (VarV) coverage rate in children aged 1–9 years in Jiangsu Province.

**Methods:**

From June to October 2016, a cross-sectional survey was conducted to collect 3631 serum samples from healthy children aged 1–9 years in Jiangsu Province. The immunoglobulin G (IgG) antibody levels of varicella were detected by enzyme-linked immunosorbent assay (ELISA).

**Results:**

The VarV coverage rate of healthy children was only 43.1% (95% CI: 41.1–44.7%). The seroprevalence after vaccination with a single dose of VarV was only 57.1%, and the overall seropositivity and geometric antibody titre (GMC) were 43.5% and 225.4 mU/ml, respectively. The seropositivity was significantly higher in girls than in boys (χ^2^ = 18.82, *P* < 0.001). The difference in seropositivity between the 5–9 age group and 1–4 age group was statistically significant (χ^2^ = 84.31, *P* < 0.001). The difference in seropositivity between different regions was statistically significant, with the highest seropositivity in the northern area, 53.7% (χ^2^ = 35.64, *P* < 0.001). The seropositivity in the group receiving one dose of VarV was significantly higher than that of the unvaccinated group (χ^2^ = 205.16, *P* < 0.001). Linear regression analysis suggested that the GMC of varicella antibodies wanes with the time since vaccination (F = 65.01, *P* = 0.002).

**Conclusion:**

The VarV coverage rate of healthy children in Jiangsu Province was low. Sero-conversion rates were also low after one dose of VarV, and the immune effectiveness of a single dose of VarV was limited. To control the spread of varicella, VarV should be included in the routine immunization program, and strengthened immunization measures for the varicella-susceptible population warrant additional consideration.

## Background

Varicella is a highly contagious disease caused by initial infection by the varicella-zoster virus [1]. Varicella is prone to spread in collective institutions, such as kindergartens and primary and secondary schools. The most effective way to reduce the incidence of varicella in children is through vaccinations with the varicella attenuated live vaccine (VarV) [2, 3]. Until recently, the coverage rate of one-dose varicella vaccine was low because it is still a voluntarily self-funded vaccine and has not been introduced to the Expanded Programme of Immunization in Jiangsu Province, China. Unvaccinated students can receive emergency vaccination for free when a varicella outbreak occurs in school [4]. Individuals aged 12 months to 12 years old are recommended to receive 1 dose of VarV by private purchase [4, 5]. Of the varicella cases in Jiangsu Province in 2016, 76.5% were among children aged 1–9 years old. Most recently, outbreaks of varicella have continued to occur, and breakthrough cases of varicella have received increasing attention and are becoming a serious public health problem [6].

To gain a comprehensive understanding of the vaccination coverage rate of children’s VarV in Jiangsu Province and to describe the profile of immunoglobulin G (IgG) levels of varicella antibodies among children, a sero-survey of varicella was conducted in healthy children in Jiangsu Province, China.

## Materials and methods

### Varicella surveillance

Surveillance and management of varicella are based on its designation as a class C infectious disease in China. All patients diagnosed with varicella by a hospital or laboratory need to be recorded in the National Notifiable Disease Reporting System, a web-based computerized reporting system. Demographic data were provided by the Jiangsu Provincial Bureau of Statistics.

### Serological survey

**Fig. 1:**
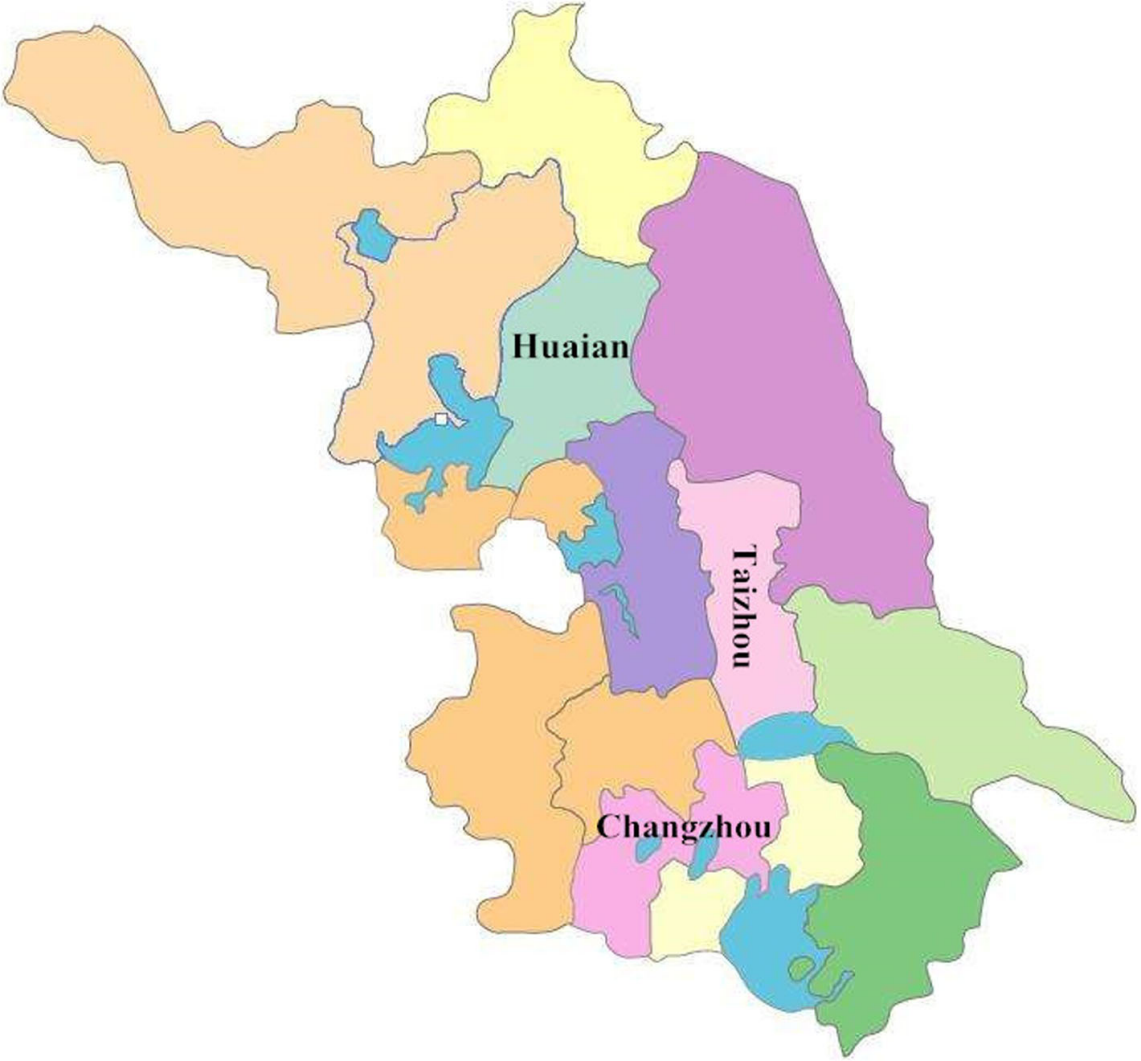
Map of Jiangsu Province; the cities randomly selected based on geographical location and economic situation are Huaian, Taizhou and Changzhou

A cross-sectional survey was conducted by stratified cluster random sampling from June to October 2016. In the first stage, the province was divided into three regions, namely, south, central and north, according to socio-economic level and geographical location. One city was randomly selected from each region as a research site: Changzhou, Taizhou and Huaian (see Fig. 1). In the second stage, the random number table method was used to select 3–5 townships in each city. In the third stage, children were recruited from the townships included in the sample. The inclusion criteria for children were as follows: (1) aged 1–9 years and had consent from a guardian for blood collection; (2) had been a local resident for at least 3 months; and (3) were in good physical health (with axillary temperature < 38 °C and without acute or chronic diseases). The exclusion criteria were as follows: (1) refused collection of venous blood; or (2) had a serious illness or other medical reasons for not participating in the study after clinical evaluation.

A total of 3709 eligible children were recruited in this study. Seventy-eight children refused to donate venous blood for unknown reasons, resulting in a total of 3631 serum samples from individuals aged 1–9 collected from January to November 2016. The study was approved by the Medical Ethics Committee of the Jiangsu Provincial Center for Disease Control and Prevention (No: SL2015-B015–02). Written informed consent was obtained from the parents/guardians of the children before enrolment. Guardians of the children were required to complete a questionnaire regarding their children’s information and immunization history, such as age, gender, date of birth, date of vaccination, and date of sampling. If the entire immunization history of a child was missing, we looked for it on the Jiangsu Provincial Immunization Information System.

### Laboratory assay

Serum samples were collected and frozen at − 70 °C until testing. Enzyme-linked immunosorbent assay (ELISA) was used to detect varicella-specific IgG antibodies; all the experiments were performed at the laboratory of the Department of the Expanded Programme on Immunization at the Jiangsu Provincial Center for Disease Control and Prevention. To avoid test bias, all detection tests were conducted by the same staff members using commercial ELISA kits from Institut Virion\Serion GmbH (SERION ELISA classic anti-varicella virus IgG, batch number: SLF.CL). The ELISA results were first expressed as optical density (OD) measurements at 402 nm and later converted to the antibody concentration values (U/ml) using software from SERIO, according to the product instructions. According to the kit instructions, the serum antibody concentration ranged from 15 to 2000 mIU/ml, the samples with an antibody concentration > 2000 mIU/ml were labelled as 2000 mIU/ml, and samples < 15 mIU/ml were labelled as 15 mIU/ml. An antibody concentration ≥ 100 mIU/ml was considered positive, and a concentration < 80 mIU/ml was considered negative. In addition, we used commercial ELISA kits from the same company, and all tests were conducted by the same staff members with strict quality control under similar laboratory conditions. Furthermore, equivocal samples (80–100 mIU/ml) were retested prior to categorization as positive or negative.

### Statistical analysis

EpiData software was used to input information from the questionnaires in duplicate, with suitable edits and validations. Geometric antibody titres (GMCs) in different groups were compared by one-way ANOVA, and multiple comparisons were performed using the SNK-q test. The antibody seroprevalence in different age groups, gender groups, region groups and immune status was compared by Pearson’s χ^2^ test. The relationship between the GMC value and vaccination duration was analysed by linear regression. All statistical tests used a two-sided α = 0.05 as the significance threshold and were performed using R 2.10.0 statistical software.

## Results

### Sero-survey of varicella in children aged 1–9 years in 2016

A total of 3631 serum samples from healthy children in Changzhou, Taizhou and Huaian were collected. Among them, 1563 children had a history of VarV vaccination, resulting in a vaccination coverage rate of 43.1% (95% CI: 41.4–44.7%). See Table 1 for details. The antibody sero-conversion rate was 57.1% (95 CI: 54.6–59.5%) after one dose of VarV. The overall seroprevalence of varicella antibodies was only 43.5% (95% CI 41.9–45.1%). The seroprevalence of varicella antibodies in the 1–4-year age group was significantly higher than that in the 5–9-year age group (52.3% vs 37.0%, *P* < 0.001). The positive rate of varicella antibodies in girls was significantly higher than that in boys (47.3% vs. 40.1%, *P* < 0.001). The seroprevalence of children with a history of immunization was significantly different from that of children without immunization (57.1% vs. 33.3%, *P* < 0.001). The positive rate of virus antibody was the highest in children in northern Jiangsu (53.7, 95% CI: 50.8–56.5%) and was significantly higher than the rates in southern Jiangsu and Suzhong (*P* < 0.001); See Table 2.

**Table 1:** Characteristics of coverage of varicella vaccine for healthy children in some areas of Jiangsu Province in 2016

Characteristics		Sample size	Number of children vaccinated	Coverage rate(%,95 CI)	χ^2^ value	*P* value
Age (year)	1–4	1543	740	47.1(44.5–49.5)	2.98	0.084
	5–9	2088	941	45.1(42.9–47.2)		
Gender	Boy	1898	1080	56.9(54.6–59.1)	60.07	< 0.001^a^
	Girl	1733	763	44.1(41.7–46.4)		
Area	Changzhou	1249	590	47.2(44.4–50.1)	107.18	< 0.001^a^
	Taizhou	1178	609	51.7(48.8–54.6)		
	Huaian	1204	582	31.7(29.1–34.4)		
Total		3631	1581	43.1(41.4–44.7)		

Attention: ^a^ means *P* value≤0.05

**Table 2:** Analysis of IgG antibody levels of varicella in children aged 1–9 years in Jiangsu Province in 2016

Characteristics		Sample size	N	Seroprevalence(%, 95 CI)	GMC(mU/ml, 95% CI)
Age(year)	1–4	1543	807	52.3(49.7–54.8)	275.2(235.4–297.6)
	5–9	2088	773	37.0(34.9–39.1)	183.7(153.2–207.9)
χ^2^/t value			84.31	3.65	
*P v*alue			< 0.001	< 0.001	
Gender	Boy	1898	761	40.1(37.9–42.3)	212.6(175.2–236.5)
	Girl	1733	819	47.3(44.9–49.6)	260.4(240.3–295.2)
χ^2^/t value			18.92	2.52	
*P v*alue			< 0.001	< 0.001	
Area	South	1249	438	35.1(32.4–37.8)	216.2(198.7–232.4)
	North	1204	646	53.7(50.8–56.5)	191.8(178.5–221.2)
	central	1178	496	42.1(39.3–44.9)	298.1(273.1–321.6)
χ^2^/F value			35.64	24.21	
*P v*alue			< 0.001	< 0.001	
Immune history	1 dose	1563	892	57.1(54.6–59.5)	294.9(265.2–330.4)
	0 dose	2068	688	33.3(30.9–35.1)	120.2(97.5–153.6)
χ^2^/t value			205.16	5.36	
*P v*alue			< 0.001	< 0.001	
Total	3631	1580	43.5(41.9–45.1)	225.4(185.4–247.6)	

Among children with a history of VarV vaccination, the longer it had been since they had received the VarV, the lower their antibody GMC value was, suggesting that the varicella antibody titre has a tendency to wane with time (F = 69.01, *P* = 0.003); See Table 3.

**Table 3:** Relationship between GMCs, seroprevalence and time since vaccination before the sero-survey

Time since vaccination (year)	Sample size	N	Seroprevalence(%, 95 CI)	GMC(95% CI, mU/ml)	Statistics	*P* value
1	48	29	60.4(45.3–74.2)	298.6 (275.2–313.5)	F = 65.01	0.002
2	130	74	56.9(47.9–65.6)	263.5(243.4–280.6)		
3	415	211	50.8(45.9–55.8)	218.7(198.6–234.7)		
4	111	52	46.8(37.3–56.6)	224.8(205.1–239.8)		
5	185	91	49.2(41.8–56.6)	164.2(124.1–185.4)		
6	604	224	37.1(33.2–41.1)	155.6(134.2–177.9)		
7	70	27	38.6(27.2–50.9)	165.3(144.2–188.9)		

In addition to the incidence of varicella and the seroprevalence of antibodies in 2016, the reported incidence of varicella was the lowest and the seroprevalence was the highest in northern Jiangsu Province. The reported incidence rate decreased with increases in the antibody positive rate.

## Discussion

Varicella is a highly contagious disease that can be transmitted through daily contact [7]. The susceptible population is concentrated in children, particularly those in nurseries, kindergartens, primary and secondary schools and other collective units [7–9]. Immunocompromised adults with varicella are more likely to experience a severe course and have serious complications [10]. Since 2015, reported varicella cases have increased in number and have become a serious public health problem, and outbreaks of varicella have been reported among highly vaccinated preschool children [5, 6, 11, 12]. The increasing number of varicella cases between 2016 and 2017 in Jiangsu Province may be the result of the continuing low vaccination coverage, which leads to an accumulation of susceptible persons. There have been many studies to date on antibody levels in varicella, but few studies on the VarV coverage rate and the attenuation of antibody levels in healthy children, which could help optimize immunization programs in China, have been performed [13]. This study conducted a cross-sectional survey on varicella IgG antibody levels in 2016 to understand the actual vaccination coverage rate of healthy children in Jiangsu Province, to comprehensively describe the immunization profile of children aged 1–9 years old based on varicella surveillance data, and to provide an immunological basis for VarV to be included in the routine immunization program.

This study found that the vaccination coverage rate of healthy children in Jiangsu Province was low (43.1%), and it was lower than the estimated coverage rate of varicella in Jiangsu Province’s vaccine management system (approximately 55–65%). The overall antibody positive rate (43.5%) and antibody GMC (225.4 mU/ ml) were lower than those reported in relevant studies in Beijing city and Shanghai city [14–16]. According to several clinical trial and observational studies in the domestic and foreign literature, relatively low vaccination rates and sero-conversion rates will lead to an increase in varicella outbreaks [17–19]. This study’s findings confirm previous reports that single-dose varicella vaccine is insufficient to provide the population with an immune barrier. Based on varicella surveillance and seroprevalence data, it was found that there was a negative correlation between the reported incidence and seroprevalence among regions, which indirectly indicated that the immunization effect of varicella vaccine could prevent the spread of varicella to a certain extent [20]. The state of the varicella epidemic is the most serious and the seroprevalence of antibody in children is the lowest in the southern Jiangsu region, meaning that the occurrence of varicella virus infection in this region can easily cause an epidemic outbreak of varicella. This study shows that the seroprevalence is significantly higher in girls than in boys, and the reported incidence rate is lower in girls than in boys. The reasons for this finding may be complicated and may include the different contact rate, different exposure levels and different asymptomatic infection rate of these children [21, 22]. By contrast, a German study showed that there was no difference between boys and girls, which may indicate cultural differences, and in-depth reasons for this discrepancy should be further explored [23].

The study showed that among 1563 children with a history of VarV vaccination, the seroprevalence of varicella antibodies was only 57.1%, which may be one of the reasons for the increase in breakthrough cases, and it also indicates that the effect of a single dose of VarV is limited. This study found that the antibody GMC values tend to wane with the time since vaccination. Several studies on school outbreaks have suggested that extended time since vaccination may be associated with the possibility of breakthrough varicella [18, 24, 25]. To better control the varicella epidemic, the need to increase immunization coverage rates with varicella vaccine among children in Jiangsu Province has also been suggested. The US Advisory Committee on Immunization Practices issued the following regulations on the second dose of varicella vaccination in 2006: it is recommended that children aged 4–6 who have received one dose of varicella vaccine be vaccinated once more. The surveillance and investigation results show that two doses of varicella vaccine are more effective in controlling the occurrence of varicella breakthrough cases [26, 27]. It is suggested that Jiangsu Province implement a two-dose varicella vaccine schedule for children and maintain high coverage of two-dose varicella vaccine for children enrolled in kindergartens and primary schools; this approach could improve protection from both primary vaccine failure and waning vaccine-induced immunity [18].

This study described the VarV coverage rate and immunization level of healthy children aged 1–9 years in some areas of Jiangsu Province using a large sample size. One limitation of this study is that it is a cross-sectional study, which provides insight only regarding the immunization strategy of varicella; evidence from prospective studies is still needed. Another limitation is that seropositivity is only a reference range for preventing varicella infection. The seropositivity can only roughly reflect the susceptibility of children to varicella virus, and the optimal age for the second varicella vaccine warrants further exploration.

## Conclusions

Overall, this study summarizes the findings of an observational population-based study of varicella immunity in children aged 1–9 years and shows that these children were at high risk of varicella infection. Waning immunity in terms of seroprevalence and GMCs was observed. The VarV coverage rate of healthy children in Jiangsu Province was low. Sero-conversion was also low after one dose of VarV was administered, and the immune effect of a single dose of VarV was limited. To better control varicella spread, VarV should be included in an expanded immunization program, and a second dose of varicella vaccine should be recommended for all children, which could compensate for the first immune failure and waning-induced immunity.

## Abbreviations

ELISA: Enzyme-linked immunosorbent assay
GMCs: Geometric antibody titres
IgG: Immunoglobulin G
VarV: Varicella attenuated live vaccine
